# Functional Consequence of *IRF6* Variants in Van der Woude Syndrome

**DOI:** 10.3390/genes17101223

**Published:** 2026-10-02

**Authors:** Mojisola Olujitan, Rose Gogal, Tamara Busch, Chloe Ovel, Anjolaoluwa Awe, Jorge Ceballos, Kikanwa Okiosor, Natalie Ibarra, Emmanuel Aladenika, Waheed Awotoye, Veronica Sule, Babatunde Faparusi, Benjamin Bass, Rishitha Gadde, Thirona Naicker, Lord J. J. Gowans, Mekonen Eshete, Wasiu Lanre Adeyemo, Aline Petrin, Martine Dunnwald, Carmen J. Buxo, Peter Mossey, Michael Schnieders, Azeez Butali

**Affiliations:** 1 Iowa Institute for Oral Health Research, University of Iowa, Iowa City, IA 52242, USA; mojisola-olujitan@uiowa.edu (M.O.); aline-petrin@uiowa.edu (A.P.); 2 Department of Oral Pathology, Radiology and Medicine, College of Dentistry, University of Iowa, Iowa City, IA 52242, USA; 3 Roy J. Carver Department of Biomedical Engineering, University of Iowa, Iowa City, IA 52242, USAmichael-schnieders@uiowa.edu (M.S.); 4 GKT School of Medical Education, King’s College London, London WC2R 2LS, UK; 5 College of Dentistry, University of Iowa, Iowa City, IA 52242, USA; 6 School of Dentistry, University of Michigan, Ann Arbor, MI 48109, USA; 7 Department of Orthodontics, College of Dentistry, University of Iowa, Iowa City, IA 52242, USA; 8 Department of General Dentistry, School of Dentistry, Creighton University, Omaha, NE 68178, USA; 9 Department of Pediatrics, University of KwaZulu-Natal, Pinetown 3605, South Africa; 10 Department of Biochemistry and Biotechnology, Kwame Nkrumah University of Science and Technology, Kumasi 00233, Ghana; ljj.gowans@gmail.com; 11 Surgical Department, School of Medicine, Addis Ababa University, Addis Ababa P.O. Box 9086, Ethiopia; 12 Department of Oral and Maxillofacial Surgery, University of Lagos, Lagos 100254, Nigeria; 13 Department of Anatomy and Cell Biology, Carver College of Medicine, University of Iowa, Iowa City, IA 52242, USA; martine-dunnwald@uiowa.edu; 14 Dental and Craniofacial Genomics Core, School of Dental Medicine, University of Puerto Rico, San Juan, PR 00936, USA; carmen.buxo@upr.edu; 15 School of Dentistry, University of Dundee, Dundee DD1 4HN, UK

**Keywords:** Van der Woude Syndrome, *IRF6*, variant effect prediction

## Abstract

Van der Woude syndrome (VWS) is the most common syndromic form of orofacial clefts (OFCs). The heritability of VWS is largely (~70%) explained by variants in the *IRF6* gene. Experimental studies to confirm genetic variant effects are essential but are time intensive and expensive. Advancing next-generation sequencing techniques has made it possible to identify genetic variation quicker than their causal effects in disease processes can be ascertained. Therefore, employing computational tools to reliably predict their causal effects is crucial for advancing translational research. We conducted Sanger sequencing of the *IRF6* gene in 46 VWS cases across 34 families to identify likely causal missense variants. We then assessed structure-informed stability changes to the IRF6 protein when these variants were incorporated into the protein, using DDGun3D. We compared DDGun3D predictions to results from previous experimental studies. Among four variants previously shown to prevent functional rescue in zebrafish, only the variant *IRF6*(NM_006147.4):c.881T>C, shown to cause rupture of zebrafish embryo, exhibited a high free energy change (∆∆G*_fold_* = 3.5 kcal/mol), indicating that substantial predicted thermodynamic destabilization does not capture all disruptive function of IRF6. Our study demonstrates the utility of a protein structure-based computational approach for functional variant interpretation, providing a complementary method for studying genetic variation. Integrating computational predictions with experimental data can enhance our understanding of genetic conditions. However, success depends on fully elucidating protein structures, which is an active area of research.

## 1. Introduction

Van der Woude syndrome (VWS) is a congenital disorder primarily presenting with distinctive lower lip pits alongside cleft lip and/or palate [1]. Affecting approximately 1 in 35,000 live births, VWS is the most common syndromic form of clefting, accounting for around 2% of all cleft lip and palate (CL/P) cases [2,3,4]. It uniquely manifests equally across sexes and is characterized by autosomal dominant inheritance with relatively high penetrance [5]. Despite this high penetrance, VWS exhibits a broad spectrum of clinical variability, from mild forms involving only lip pits to severe clefts involving the lip and/or palate. Affected individuals may display any combination of characteristic features or, in some cases, no detectable abnormalities, underscoring the complex, heterogeneous expression patterns observed in this syndrome [6].

The genetic foundation of VWS lies predominantly in mutations affecting the Interferon Regulatory Factor 6 (*IRF6*) gene, located on chromosome 1q32 [1,7]. The *IRF6* gene encodes a transcription factor crucial for orofacial, skeletal, and epidermal development [8,9]. It regulates processes essential for the formation of the lip and palate with a required threshold of expression necessary for optimal development [10,11]. Within the IRF6 protein, the DNA-binding and protein-binding domains are especially critical for its biological function [12,13]. Alterations within these domains, particularly in exons 3, 4, and 7–9 have been identified as central contributors to the VWS phenotype [14]. Roughly 70% of all *IRF6* mutations associated with VWS occur in these domains [15]. Additionally, mutations in *IRF6* have been implicated in non-syndromic forms of cleft lip and/or palate highlighting a genetic overlap between these two categories, as well as evidence of a role in severity of phenotypic expression [16,17]. This overlap and variability in presentation represents a complex relationship between gene function, regulatory factors, and the environment [18,19]. Therefore, pinpointing directly causal genetic variants in *IRF6* can translate into useful diagnostic, risk assessment, and therapeutic strategies.

*IRF6* does not function in isolation; it operates as part of a regulatory network with other genes involved in craniofacial development [17,20]. Understanding the genetic underpinnings of VWS requires sophisticated gene sequencing studies [4,21], which have identified various mutations within *IRF6*. However, the role of these mutations in VWS and other cleft disorders remains unclear [22]. The majority of disease-causing mutations (~51.7%) identified in VWS are missense mutations, i.e., point amino acid substitutions, which may or may not affect protein stability and function [2]. The consequences of these substitutions may not necessarily be a detectable phenotype [23]; hence, reliable categorization of these mutations is important for a deep understanding of their contribution to disease.

Experimental studies to confirm the etiology of VWS and CL/P are costly and time-consuming. Therefore, computational tools that reliably predict the pathogenicity of observed mutations within *IRF6* and other relevant genes would be highly beneficial to the research and clinical fields [24,25]. Traditionally, bioinformatic tools, such as Sorting Intolerant from Tolerant (SIFT), Polymorphism Phenotyping (PolyPhen2), Combined Annotation Dependent Depletion (CADD), and Rare Exome Variant Ensemble Learner (REVEL), amongst others, have been used to assess the likely impact of mutations on protein function [26,27,28,29]. These tools, however, often yield conflicting results and rely on limited experimental data.

Deep learning models to predict variant effects are attempting to address these shortcomings. Unsupervised models, which do not rely on annotated variant datasets, instead learn directly from amino acid sequence patterns, and distributions and have been shown to effectively predict the impact of missense variants based on the natural evolution of protein sequences [30]. However, they still lack the detailed structural insights provided by cutting-edge tools like AlphaFold3. AlphaFold3, a breakthrough in protein structure prediction, models protein folding with accuracy, capturing complex interactions that are crucial for understanding the contextual effects of mutations in several genes [31].

The application of these multi-level computational and structural approaches is critical to refining our understanding of VWS and advancing both the diagnosis and potential therapeutic options for affected individuals. By combining genetic, bioinformatic, and structural biology insights, researchers are better equipped to decode the nuances of complex genetic conditions, such as VWS, moving us closer to improved predictive models for highly variable clinical presentation.

The aim of this study is to demonstrate the utility of a protein structure-based computational approach for interpreting genetic variants. The Gibbs free energy change (ΔΔG*_fold_*) between wild-type and mutant proteins is a promising direction toward understanding protein function as it translates into phenotypic expression. We employed DDGun3D in analyzing the free energy change in protein structure stability of *IRF6* mutations identified in VWS [32].

## 2. Methods

### 2.1. Sequencing

Saliva and cheek-swab samples were obtained from clinically diagnosed VWS probands and their first- and second-degree relatives from thirty-four families recruited from Nigeria, Ethiopia, Ghana, South Africa, and Puerto Rico. Ethical approval was obtained from all study sites and the primary site—the University of Iowa (IRB approval number: 201101720); Lagos University Teaching Hospital Idi-Araba, Lagos (IRB approval number: ADM/DCST/HREC/VOL.XV/321); the Obafemi Awolowo University Teaching Hospital Ile-Ife (IRB approval number: ERC/2011/12/01); Kwame Nkrumah University of Science and Technology (IRB approval number: CHRPE/RC/018/13); Addis Ababa University (IRB approval number: 003/10/surg); the University of Puerto Rico (IRB approval number: 0640111); and Kwazulu-Natal University (IRB approval number: BE309/18). We performed Sanger sequencing on all exons of the *IRF6* gene on each obtained sample. Details of the primer sequences used to amplify the exonic regions 1–9 of the *IRF6* gene have been previously published [14]. The complete set of identified *IRF6* variants and available clinical phenotypes is provided in Supplemental Table S1.

### 2.2. Protein Structure

**Figure 1:**
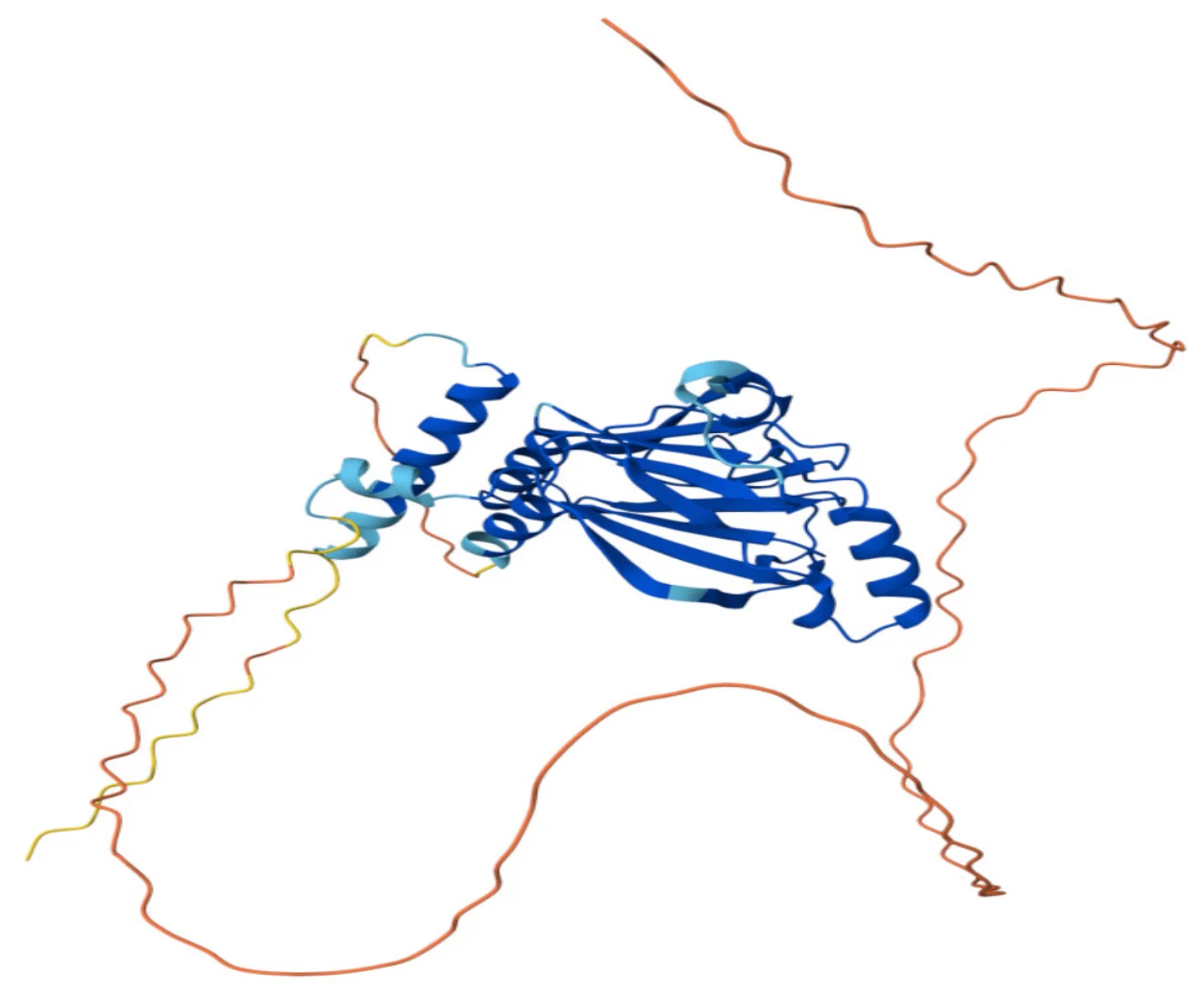
IRF6 structure. Predicted human IRF6 from AlphaFold3 [31]—demonstrates the structural context in which the analyzed variants reside. This structural context is directly relevant to the free energy analyses performed by DDGun3D, as the tool requires a three-dimensional protein model as input. AlphaFold3 uses a pLDDT—predicted Local Distance Difference Test—score which represents a per-residue confidence metric with a 0–100 range. Scores >90 indicate very high confidence (dark blue), scores at 70–90 indicate confident predictions (light blue), scores at 50–70 indicate low confidence (yellow), and scores <50 indicate very low confidence (orange).

The putative structures of the IRF6 protein were obtained from the *AlphaFold3* server (https://alphafoldserver.com/ accessed on 4 July 2024) for both isoforms of the protein in the NCBI database (https://www.ncbi.nlm.nih.gov/protein/ accessed on 4 July 2024) (Figure 1). The two IRF6 protein isoforms were evaluated independently. Variants were mapped to the corresponding protein sequence prior to structure-based analysis. Where a genomic variant generated different protein-level annotations across isoforms, the applicable isoform-specific substitutions were analyzed separately. Some limitations of the AlphaFold3 algorithm include the propensity to predict structures with overlapping atoms, thereby creating hallucinations and disordered regions. This results in an unrealistic protein structure [31]. This necessitates the use of Force Field X (FFX) to refine the AlphaFold3 predictions by combining advanced energy functions with experimental data. FFX helps resolve clashes while optimizing the protein backbone [33]. AlphaFold3 relies heavily on neural network predictions with local minimization using the Amber fixed charge force field, and FFX uses the Atomic Multipole Optimized Energetics for Biomolecular Application (AMOEBA) polarizable force field. AMOEBA describes the effects of charge using multipole electrostatics and polarization effects in the protein environment, enhancing structural precision [34]. We performed local minimization using the limited-memory Broyden–Fletcher–Goldfarb–Shanno (L-BFGS) method to resolve backbone clashes. We then applied a many-body expansion to optimize side-chain positions in relation to the backbone, resolving steric clashes and improving overall structure [35].

### 2.3. Variant-Effect Predictions

Traditional in silico predictive tools for missense mutation effect predictions were used to obtain functional predictions of the effects of top variants; SIFT, PolyPhen2, CADD, and REVEL. DDGun3D, an untrained method for predicting protein stability change that includes structural and sequence information, was then used to predict the protein stability changes in the optimized protein structure [32]. It incorporates the three-sequence-based DDGun scores together with an additional structure-based score (sBV), which evaluates differences in interaction energy between the wild-type and variant residues and their local structural environment within a 5 Å radius using the Bastolla–Vendruscolo statistical potential [32,36]. DDGun3D reports predicted changes in unfolding free energy in kcal/mol. For presentation and interpretation, these values were converted to the ΔΔ*G_fold_* convention, defined as the difference in folding free energy between the variant and wild-type proteins (ΔG_variant_ − ΔG_WT_); therefore, the sign of the raw DDGun3D output was inverted. Under this convention, positive ΔΔG*_fold_* values indicate predicted destabilization, whereas negative ΔΔG*_fold_* values indicate predicted stabilization. Large positive changes (ΔΔG*_fold_* > 1 kcal/mol) indicate a destabilizing mutation while negative changes indicate a stabilizing mutation.

### 2.4. Plausibility Assessment Through Experimental Studies

A study published in 2017 by Li et al. had confirmed the consequences of reported mutations in *irf6* using the zebrafish model [37]. In this study, human *IRF6* variants were mapped to the corresponding location in zebrafish *irf6* cDNA. Zebrafish *irf6^−/−^* embryos were generated using CRISPR and were demonstrated to exhibit early-stage embryonic periderm disruption resulting in failure to initiate epiboly. To determine the functional effect of previously published missense human IRF6 variants (Table 1), the authors employed silico predictive tools, SIFT and PolyPhen2 and compared the results to experimental validation of these variants. The experimental phase involved the microinjection of missense gene variants (zebrafish and human mRNA) at the single-cell stage of the *irf6^−/−^* embryo zebrafish to assess the ability of these variants to rescue or rupture the abnormal periderm maturation and epiboly phenotype. Variants that resulted in reversal of the abnormal phenotype were said to result in a “RESCUED” phenotype, while variants that failed to reverse the phenotype were categorized as “RUPTURED”. These data are summarized in Table 1 below. We then used this data as a comparative benchmark for predictions from DDGun3D. The comparison was done to assess the accuracy of DDGun3D predictions of variants identified in our case cohort.

## 3. Results

Variants were annotated with their amino acid changes and genomic location and predictions from in silico predictive tools, SIFT, PolyPhen2, CADD, and REVEL, which evaluated potential pathogenicity (summarized in Table 2). We then applied DDGun3D to predict the structural effects of these variants on protein stability. The majority of the variants identified in this cohort were consistently predicted to be damaging by these tools. However, some inconsistencies in the predictions were observed. rs2077901587 was tolerated by SIFT, while other tools (PolyPhen2 and CADD; score, 25.7) flagged it as potentially damaging. Combining PolyPhen2 and CADD with a high REVEL score of 0.965 confirmed a strong likelihood of functional impact. Similarly, predictions for rs187015884 were mixed, with a deleterious annotation from SIFT but benign from PolyPhen2. The CADD (21.7) and REVEL (0.239) scores suggest a lower likelihood of pathogenicity.

**Figure 2:**
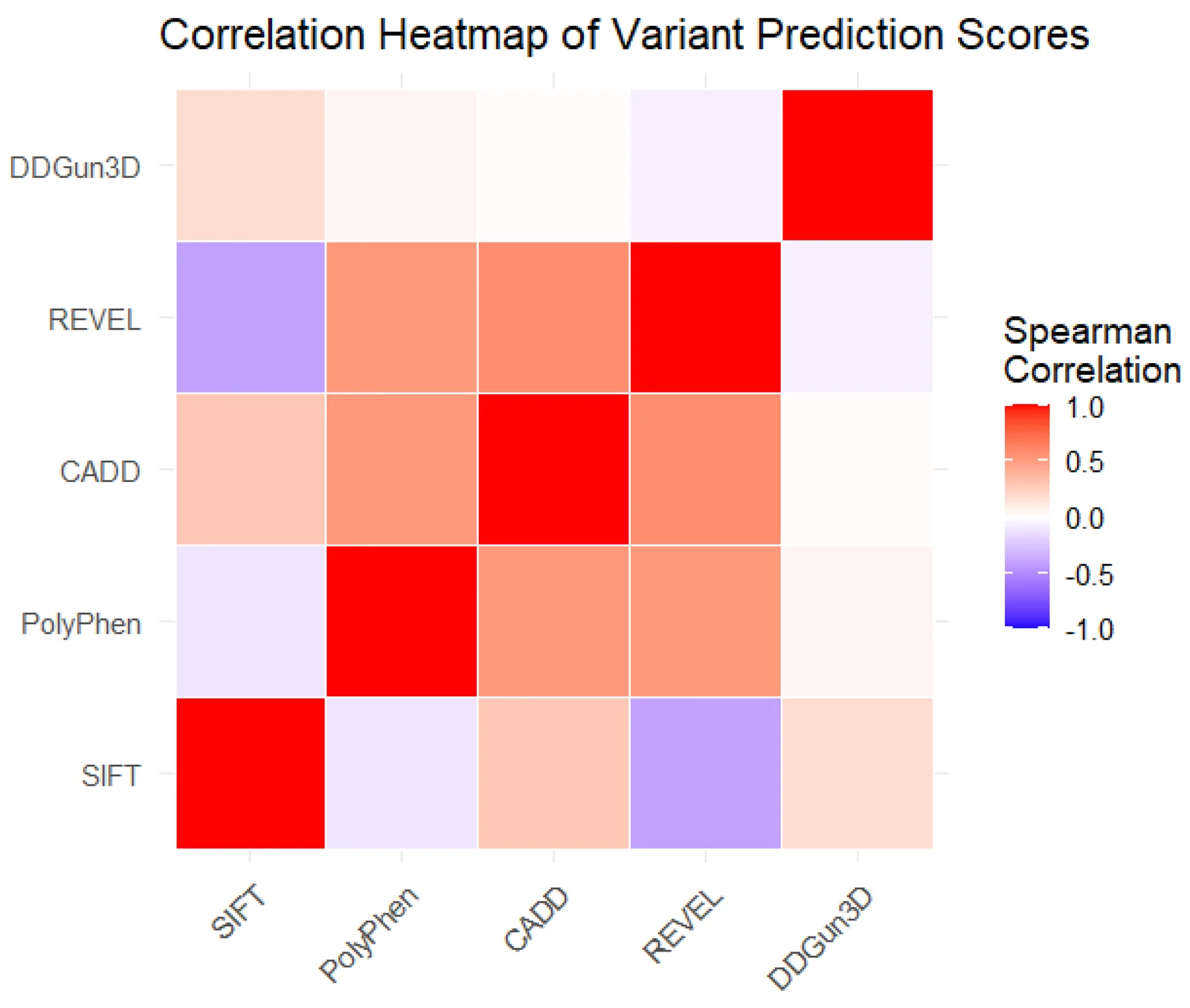
Correlation heatmap of variant pathogenicity predictions from in silico predictive tools. Spearman correlation heatmap of variant-effect prediction. The heatmap shows pairwise correlation between variant-effect prediction tools: SIFT, PolyPhen2, CADD, REVEL and DDGun3D. Red indicates stronger correlations, while blue is indicative of weaker correlations. SIFT and PolyPhen2 scores were converted to a numeric scale to computation correlation scores. SIFT, Sorting Intolerant from Tolerant; PolyPhen2, Polymorphism Phenotyping; CADD, Combined Annotation Dependent Depletion; REVEL, Rare Exome Variant Ensemble Learner. DDGun3D’s lack of correlation with SIFT, PolyPhen and CADD demonstrates that it captures a dimension of variant impact and structural destabilization that is not captured by these tools. This weak correlation supports its use as a complementary approach to variant-effect prediction.

Most variants yielded results which suggest minimal structural impact (ΔΔ*G_fold_*); the variant c.960G>C (p.Lys225Asn) in the protein-binding domain of *IRF6* showed a ΔΔ*G_fold_* of 0.1 kcal/mol, indicating minimal structural destabilization. Figure 2 shows a correlation heatmap of variant scores across each tool listed in Table 2. SIFT, PolyPhen, CADD, and REVEL show moderate-to-strong correlation, reflecting shared features or overlapping training data. In contrast, DDGun3D shows weak or inverse correlation with the others, as it captures different information on variant impact. Due to the differences in the scoring scales across the different tools, the Spearman correlation method was used to assess the relationship between scores from each tool. Categorical predictions (SIFT and PolyPhen2) were converted to a numerical scale to compute scores.

Variants examined from the Li et al. experimental studies were used as a comparative benchmark **(**Table 3) [37]. The variant c.298A>G (p.Thr100Ala) had a ΔΔ*G_fold_* of 0.2 kcal/mol, suggesting minimal structural destabilization, and the variant Leu294Pro showed a ΔΔG*_fold_* of 3.5 kcal/mol, associated with significant structural destabilization. In silico predictions of these variants using SIFT and PolyPhen2 in the Li et al. study failed to consistently predict the effect of the amino acid substitutions in these mutations, demonstrating the limitation of these tools.

## 4. Discussion

We analyzed several missense variants in the *IRF6* gene (NM_006147.4), a key gene implicated in craniofacial development [1,38,39], to predict their functional impact using computational tools. The utility of DDGun3D is specifically tailored to evaluate single point mutations, limiting our analysis to missense substitutions. We first provided predictions generated by SIFT, PolyPhen2, CADD, and REVEL. Notably, most of these predictive assessments demonstrated a high degree of concordance. To support confidence in the DDGun3D predictions applied to our cohort variants, we applied the tool to a subset of *IRF6* variants from Li et al. for which experimental functional outcomes were available and compared the resulting ΔΔG*_fold_* values against those published for the zebrafish phenotype. This comparison served as a plausibility check rather than a comprehensive validation exercise; the primary application of DDGun3D in this study remains the missense variants identified in our VWS patient cohort. The twelve variants selected for this comparison were missense variants subjected to mRNA dosage titration experiments in the Li et al. study, representing the most rigorously confirmed functional classifications in that dataset.

Arg45Gln, Arg45Trp, Gly70Arg, and Val274Ile demonstrated a rescued zebrafish experimental model in the Li et al. study. Conversely, Lys66Thr, Arg84Cys, Leu294Pro, and Gly325Glu resulted in a ruptured zebrafish embryo, reflecting a deleterious impact on protein stability or function. There was alignment of our DDGun3D findings for select mutations, including Thr100Ala (ΔΔ*G_fold_* = 0.2 kcal/mol) and Leu294Pro (ΔΔ*G_fold_* = 3.5 kcal/mol), with the zebrafish experimental outcomes. This concordance supports the plausibility of applying DDGun3D to our cohort variants, though it does not constitute formal validation of the tool across the full IRF6 variant spectrum.

An important limitation of the DDGun3D analysis is that predicted thermodynamic destabilization did not consistently correspond with previously reported functional impairment. Of the four variants that failed rescue in the zebrafish assay, only p.Leu294Pro (ΔΔ*G_fold_* = 3.5 kcal/mol) exceeded the 1 kcal/mol destabilization threshold. Lys66Thr (0.6 kcal/mol), Arg84Cys (0.9 kcal/mol), and Gly325Glu (0.7 kcal/mol) all scored below this threshold despite confirmed functional deleteriousness. This indicates that thermodynamic destabilization of the folded protein is not the sole mechanism through which these variants exert pathogenic effects. Alternative mechanisms likely include disruption of protein–protein interaction surfaces without global unfolding, effects which are not captured by ΔΔ*G_fold_* alone. Protein structural stability is a multidimensional property, and ΔΔ*G_fold_* assesses only the thermodynamic folding component. We recommend that DDGun3D predictions be used in conjunction with, rather than in replacement of, sequence-based pathogenicity predictors. A combined scoring framework where variants that exceed pathogenicity thresholds in at least two independent lines of evidence is more likely to be sensitive across pathogenic variants with varying mechanisms of effect. This limitation also reinforces the ACMG principle that computational evidence constitutes a single supporting criterion and should not be treated as independent evidence.

Several mutations in the *IRF6* gene have been associated with VWS and non-syndromic CL/P [1,2,14,17,40,41]. However, the exact mechanisms by which these mutations lead to the observed phenotypes are not fully understood. For the novel variants identified in our VWS-case cohort, it is imperative to note that our DDGun3D predictions are not indicative of confirmed functional consequences. The ΔΔG*_fold_* values reported for these cohort variants represent indicators of potential structural impact which undoubtedly require further investigation. Gaining insights into gene function through the resultant protein structure and function is a crucial starting point [42,43]. This involves interpreting how specific mutations affect protein stability, folding, and interactions [44], which can help elucidate their impact on orofacial development. Efforts in structural biology to build models of protein structures have typically been based on assessing putative structures based on homology and pairwise atomic bonds which often result in static models missing the full biological protein properties [31,45]. Recent advances involve incorporating AMOEBA force field models to solve these protein structures for a more realistic protein structure which helps translate into more meaningful interpretations of the effect of amino acid substitutions on protein function [34,35].

As genetic sequencing studies have become increasingly more affordable, the identification of potentially causal genetic variations has increased significantly. However, this rapid increase presents a challenge; the ability to quickly determine the functional significance of these variations has not kept pace. In fact, a large number of identified variants are classified as Variants of Uncertain significance [46]. Even though multiple in silico tools, like SIFT, PolyPhen2, CADD, and REVEL, ensure a robust assessment of variant pathogenicity due to variations in their goals and training sets, reports are often contradictory. The capacity of DDGun3D to generate quantitative predictions of folding free energy changes (ΔΔG*_fold_*) offers an additional layer of structural insight. SIFT, PolyPhen-2, CADD, and REVEL showed moderate-to-strong correlations, reflecting overlap in the information incorporated into their variant-effect predictions. In contrast, DDGun3D showed weak or inverse correlations with these scores. Unlike these broader variant-effect predictors, DDGun3D directly estimates the predicted effect of an amino-acid substitution on protein thermodynamic stability (ΔΔG*_fold_*), thereby providing a distinct biophysical measure that can complement these predictions. The observed weak correlations are consistent with these methodological differences but do not independently establish improved variant-classification performance.

The structural destabilization observed in pathogenic variants such as Leu294Pro (ΔΔG*_fold_* = 3.5 kcal/mol) underscores the importance of protein folding in maintaining *IRF6* functionality. Proline is particularly unique in its rigidity and inability to donate protons; this has important ramifications for protein folding and binding [47,48]. However, the present analysis did not establish that these properties specifically account for the predicted destabilization of this residue change. The downstream molecular consequences of these predicted stability changes were not directly evaluated and require further functional investigation. Such studies could determine whether altered protein stability affects processes important to IRF6 function, including DNA binding or protein interactions [12]. We were unable to demonstrate similar structural implications for the variants identified in our cohort, in part due to some of the unsolved regions in the putative structure of IRF6. Because DDGun3D depends on the underlying three-dimensional model, predictions involving residues located within low-confidence regions of the AlphaFold3-predicted structure should be interpreted with additional caution. FFX refinement does not eliminate uncertainty regarding the native conformation of these regions.

Our findings highlight the potential of DDGun3D and similar tools. DDGun3D provides a structure-informed estimate of changes in protein thermodynamic stability, representing a distinct biophysical measure from the broader variant-effect predictions generated by SIFT, PolyPhen-2, CADD, and REVEL. This information may be considered alongside genetic, functional, and other computational evidence but is not intended as an independent determinant of pathogenicity. It is, however, a valuable complement to traditional genetic studies. By combining computational structural and biophysical predictions with sequencing data, we can gain deeper insights into the genetic architecture of human disease and build frameworks necessary for risk assessment and genetic counselling where they were previously not possible.

Predicting the structural consequences of *IRF6* variants may provide additional information for interpreting their potential effects on protein function. In this study, DDGun3D provided a structure-informed estimate of thermodynamic stability that could be considered alongside genetic, functional, and other computational evidence. However, the discordance observed between predicted destabilization and zebrafish functional outcomes demonstrates that thermodynamic stability represents only one dimension of variant effect and should not be interpreted independently as evidence of pathogenicity.

This study illustrates both the potential and current limitations of incorporating predicted protein structure into variant effect evaluation. While focused on IRF6, the methodology may be applicable to other proteins for investigating the biophysical consequences of amino acid substitutions. Continued improvements in these approaches could significantly advance structural genomics, enabling more accurate predictions of variant effects across diverse biological contexts. Protein structure determination is anessential and evolving area of research that continues to improveour ability to generate accurate protein models and better understand protein function [49,50].

## Figures and Tables

**Table 1:** Functional characterization of IRF6 mutations in zebrafish (Li et al. [37]).

Amino Acid Change	Human	Zebrafish	Mutation	Result	ACMG Classification
Arg45Gln	R45Q	R45	c.134G>A	Rescued	Uncertain
Arg45Trp	R45W	R45	c.133C>T	Rescued	Uncertain
Gly70Arg	G70R	G70	c.208G>C	Rescued	Uncertain
Val274Ile	V274I	V297	C.820G>A	Rescued	Benign
Arg400Gln	R400Q	R423	c.1199G>A	Rescued	Likely pathogenic
Arg9Trp	R9W	R9	c.25C>T	Rescued	Likely pathogenic
Asn88His	N88H	N88	c.262A>C	Rescued	Likely pathogenic
Thr100Ala	T100A	T100	c.298A>G	Rescued	Likely pathogenic
Lys66Thr	K66T	K66	c.197A>C	Ruptured	Uncertain
Arg84Cys	R84C	R84	c.250C>T	Ruptured	Pathogenic
Leu294Pro	L294P	L317	c.881T>C	Ruptured	Pathogenic
Gly325Glu	G325E	G348	c.974G>A	Ruptured	Likely pathogenic

ACMG; American College of Medical Genetics and Genomics.

**Table 2:** Computational characterization of *IRF6* mutations from cohort data.

Amino Acid Change	Human	Mutation	Base-Pair Location	rs Identifier	SIFT	PolyPhen2	CADD	REVEL	ΔΔG*_fold_* (kcal/mol)
p.(Arg84Leu)	R84L	*IRF6*(NM_006147.4):c.251G>A>T	chr1:209796476	rs121434227	Deleterious	Probably Damaging	33	0.975	0
p.(Lys320Asn)	K320N	*IRF6*(NM_006147.4):c.960G>C	chr1:209790595	-	Deleterious	Probably Damaging	22.6	0.728	0.1
p.(Lys225Asn)	K225N	*IRF6*(NM_001206696.2):c.675G>C	chr1:209790595	-					0.1
p.(Lys342Glu)	K324E	*IRF6*(NM_006147.4):c.1024A>G	chr1:209790531	-	Deleterious	Probably Damaging	26.5	0.752	0.3
p.(Gly65Glu)	G65E	*IRF6*(NM_006147.4):c.194G>A	chr1:209796533	rs2077902309	Deleterious	Probably Damaging	29	0.898	0.9
p.(Lys109Thr)	K109T	*IRF6*(NM_006147.4):c.326A>C	chr1:209796401	rs2077901587	Tolerated	Probably Damaging	25.7	0.965	0.1
p.(Phe36Tyr)	F36Y	*IRF6*(NM_006147.4):c.107T>A	chr1:209801307	rs2077940279	Deleterious	Probably Damaging	31	0.914	1.9
p.(Arg6His)	R6H	*IRF6*(NM_006147.4):c.17G>A	chr1:209801397	rs751967341	Deleterious	Probably Damaging	34	0.773	0
p.(Gln438Leu)	Q438L	*IRF6*(NM_006147.4):c.1313A>T	chr1:209788511	rs753818978	Deleterious	Probably Damaging	26.3	0.684	0.8
p.(Ile363Met)	I363M	*IRF6*(NM_006147.4):c.1089A>G	chr1:209789757	rs187015884	Deleterious	Benign	21.7	0.239	0.2
p.(Arg84Cys)	R84C	*IRF6*(NM_006147.4):c.250C>T	chr1:209796477	rs121434226	Deleterious	Probably Damaging	29.3	0.918	0.6

SIFT, Sorting Intolerant from Tolerant; PolyPhen2, Polymorphism Phenotyping; CADD, Combined Annotation Dependent Depletion; REVEL, Rare Exome Variant Ensemble Learner; Gibbs Free Folding Energy Change, ΔΔG*_fold_*.

**Table 3:** Computational characterization of IRF6 mutations in zebrafish (Li et al. [37]).

Amino Acid Change	Human	Zebrafish	Mutation	Result	ΔΔG*_fold_* (kcal/mol)
Arg45Gln	R45Q	R45	c.134G>A	Rescued	0.1
Arg45Trp	R45W	R45	c.133C>T	Rescued	0.1
Gly70Arg	G70R	G70	c.208G>C	Rescued	0
Val274Ile	V274I	V297	C.820G>A	Rescued	0.1
Arg400Gln	R400Q	R423	c.1199G>A	Rescued	0.4
Arg9Trp	R9W	R9	c.25C>T	Rescued	0
Asn88His	N88H	N88	c.262A>C	Rescued	0
Thr100Ala	T100A	T100	c.298A>G	Rescued	0.2
Lys66Thr	K66T	K66	c.197A>C	Ruptured	0
Arg84Cys	R84C	R84	c.250C>T	Ruptured	0.6
Leu294Pro	L294P	L317	c.881T>C	Ruptured	3.5
Gly325Glu	G325E	G348	c.974G>A	Ruptured	0.7

Gibbs free folding energy change, ΔΔG*_fold_*; scores > 1 are indicative of destabilizing mutations.

## Data Availability

This study includes both primary and secondary data sources. Secondary data were obtained from a previously published study, with appropriate citations provided in the manuscript. Access to these data may be subject to the data use agreements and/or institutional policies of the authors of the originating study. Data generated from the Sanger sequencing of the VWS cohort reported in this study are available upon reasonable request and subject to both institutional and ethical guidelines. The IRF6 protein structures analyzed as part of this study are available and may be downloaded from the publicly available AlphaFold3 server (https://alphafoldserver.com/ (structures downloaded on 4 July 2024)). Scripts used for data processing and analysis are available at GitHub repositories for Force Field X (https://github.com/SchniedersLab/forcefieldx, accessed on 4 July 2024, v1.0.0) and DDGun3D (https://github.com/biofold/ddgun, accessed on 4 July 2024, v2).

## Supplementary Materials

The following supporting information can be downloaded at: https://www.mdpi.com/article/10.3390/genes17101223/s1, Table S1: Clinical phenotypes of affected individuals included in the study.
