# Supplementary material for: Functional Consequence of IRF6 Variants in Van der Woude Syndrome
Source: Genes (Basel). 2026 Oct 2;17(10):1223. doi: 10.3390/genes17101223 (PMC13704330; doi:10.3390/genes17101223)
Supplement: Supplementary file 1 [file genes-17-01223-s001.zip › genes-4569028-supplementary.pdf]

Supplementary Table S1. Clinical phenotypes of affected individuals included in the study

ID	Cleft phenotype	Cleft description
1	CLP	Bilateral CL; bilateral CP
2	CLP	Left unilateral complete CL; hard and soft CP; complete left alveolar cleft
3	CPO	Untyped CP
4	CL	Right unilateral incomplete CL
5	CLP	Bilateral complete CL; hard and soft CP
6	CPO	Hard and soft CP
7	CL	Left unilateral incomplete CL
8	CPO	Soft CP
9	CLP	Right unilateral complete CL; soft CP
10	CPO	Soft CP
11	CL	Untyped CL
12	CL	Right unilateral complete CL
13	CLP	Bilateral complete CL; complete CP
14	CLP	Bilateral complete CL; complete CP
15	CL	Right unilateral incomplete CL
16	CLP	Bilateral complete CL; complete CP
17	CPO	Hard and soft CP
18	CLP	Bilateral complete CL; complete CP
19	CLP	Bilateral complete CL; hard and soft CP; complete bilateral alveolar cleft
20	CL	Bilateral complete CL; complete bilateral alveolar cleft
21	CLP	Bilateral CL; CP
22	CLP	Bilateral complete CL; hard and soft CP
23	CLP	Bilateral complete CL; complete CP
24	CPO	Hard CP
25	CLP	Bilateral complete CL; complete CP; bifid uvula
26	CLP	Bilateral complete CL; complete CP; bifid uvula
27	CPO	Incomplete submucous CP with bifid uvula
28	CLP	Left unilateral complete CL; complete CP; bifid uvula
29	CL	Bilateral complete CL
30	CL	Bilateral complete CL
31	CLP	Bilateral CL; hard and soft CP
32	CLP	CL; CP
33	CPO	Soft CP
34	CPO	CP, details not specified
35	CPO	Soft CP
36	CLP	Bilateral complete CL; complete CP
37	CLP	Left unilateral CL; CP
38	CLP	CL; CP
39	CL	Right unilateral CL involving alveolus

40	CL	Left unilateral CL involving alveolus
41	CLP	Left unilateral complete CL; hard and soft CP; complete left alveolar cleft
42	CPO	Soft CP
43	CPO	Soft CP
44	CLP	Bilateral complete CL; hard and soft CP; complete bilateral alveolar cleft
45	Unknown	Cleft type not documented
46	CLP	Bilateral CL; CP

CL, cleft lip; CLP, cleft lip and palate; CPO, cleft palate only; CP, cleft palate; VPI, velopharyngeal insufficiency.
